# Health Related Quality of Life in Interstitial Lung Disease: Can We Use the Same Concepts Around the World?

**DOI:** 10.3389/fmed.2021.745908

**Published:** 2021-10-06

**Authors:** Kerri I. Aronson, Atsushi Suzuki

**Affiliations:** ^1^Division of Pulmonary and Critical Care Medicine, Weill Cornell Medicine, New York, NY, United States; ^2^Department of Respiratory Medicine, Nagoya University Graduate School of Medicine, Nagoya, Japan

**Keywords:** HRQOL—health-related quality of life, interstitial lung disease, global, cross cultural adaptation, linguistic validation

## Abstract

Health-Related Quality of Life (HRQOL) is increasingly viewed as an important patient-centered outcome by leading health organizations, clinicians, and patients alike. This is especially true in the interstitial lung disease community where patients often struggle with progressive and debilitating disease with few therapeutic options. In order to test the effectiveness of new pharmacologic therapies and non-pharmacologic interventions globally in ILD, this will require expansion of clinical research studies to a multinational level and HRQOL will be an important endpoint to many. In order to successfully expand trials across multiple nations and compare the results of studies between different communities we must recognize that there are differences in the concepts of HRQOL across the world and have strategies to address these differences. In this review, we will describe the different global influences on HRQOL both generally and in the context of ILD, discuss the processes of linguistic translation and cross-cultural adaptation of HRQOL Patient Reported Outcome Measures (PROMs), and highlight the gaps and opportunities for improving HRQOL measurement in ILD across the world.

## Introduction

Health-related quality of life (HRQOL), or one's quality of life as it relates to health status or disease, is increasingly recognized as an important patient centered-outcome by leading health organizations (1, 2)^1^. HRQOL is a subjective, dynamic, and multidimensional concept that includes domains representative of an individual patients' goals, values, and beliefs (3, 4). Over the past several decades, various conceptual models of HRQOL have contributed to our study of HRQOL in human disease (2, 5–7). These models provide an essential structure for conceptualization of HRQOL, including both the positive and negative aspects, and are often used as a guide for research and practices that promote improved HRQOL in different populations of interest (8). HRQOL frameworks most commonly focus on the physical and psychosocial impacts of health or disease on an individual's ability to live what they consider to be a fulfilling life (9). HRQOL amongst those who share the same or different chronic diseases is often very personal and subjective. This subjectivity will vary even more depending on a person's cultural background and environment. The various domains of HRQOL (e.g., psychosocial, physical etc.) that we intend to measure therefore should ideally be considered in the context of an individual's culture and value system (10, 11). This adds a level of complexity to measurement of HRQOL as we are compelled to recognized that these constructs will differ across different cultural, religious, and socioecological contexts (12). The processes of linguistic and cross-cultural adaptation have allowed for improved measurement of HRQOL across different cultures and languages.

During the past decade, HRQOL has gained much traction as a priority endpoint in the Interstitial Lung Disease (ILD) community. ILD is a group of heterogeneous parenchymal lung diseases with various clinical courses, many of which may be progressive, fibrotic, life altering, and eventually fatal (13, 14). Patients and ILD experts alike have vocalized the importance prioritizing HRQOL as a top area of focus in research studies and clinical practice (15, 16). Though a few therapies are documented to slow progression of disease [as measured by forced vital capacity (FVC)] in idiopathic pulmonary fibrosis (IPF) and the progressive fibrotic form of other ILDs, there is now much interest in how our interventions effectively slow deterioration in HRQOL (17, 18).

Patient Reported Outcome Measures (PROMs) that measure HRQOL gather information directly from the patient (without interpretation by a clinician or anyone else) about their perspective of the quality of their life in the context of their disease and it's treatments (19, 20). There are several PROMs that have undergone validation testing to measure HRQOL in ILD. The most commonly used instruments in the past few decades were originally intended for use in other respiratory diseases, while a handful of newer instruments have been developed for use specifically in ILD and pulmonary fibrosis (21). These “condition” or “disease-specific” PROMs are intended to capture more nuanced information about the impact of living with ILD that is most pertinent to patients with this particular chronic respiratory disease (e.g., breathlessness, cough, fatigue, aspects of psychological well-being) (22). Despite the ILD-targeted items in these instruments, one must be cognizant of the interpretation of the wording of these items for those from other cultures or countries in which the instrument was not developed. For example, dyspnea, or breathlessness is a common ILD symptom that impacts HRQOL. There are various qualitative aspects of this symptom are interpreted differently across different languages and cultures (23–25).

Here we introduce the concept of measuring HRQOL around the world, and as it pertains to specifically to ILD with a focus on linguistics, regional and environmental factors, health literacy and health-care systems, and race, ethnicity, religion and spirituality. We will describe the process of cross-cultural adaptation, the work that has been performed to cross-culturally validate PROMs in ILD, and the potential challenges and opportunities for the future study of HRQOL in ILD on a global scale.

## Global Influences on HRQOL

HRQOL generally reflects each individual's perspective on their own health and is widely accepted as one of the most important patient-centered outcomes. HRQOL measures the impact a chronic disease and its treatment have on several domains of one's life and is largely influenced by cultural and spiritual backgrounds. Therefore, it is expected that the concept of HRQOL will differ across communities within a nation, as well as between countries. Given the growing number of international clinical trials and large population health studies it is increasingly important to recognize the global factors that influence accurate measurement of HRQOL, and how to potentially address them. This section focuses on general considerations for assessing HRQOL in chronic illness, which is pertinent when we consider measurement in ILDs.

### Language Diversity

Linguistic differences are an important consideration when measuring HRQOL. Historically, most HRQOL instruments have been developed in the English Language. Over the past few decades, various HRQOL scales have been internationally translated and standardized across different languages. Translation approaches are traditionally performed by qualified academicians or language experts. With advancements in technology, online translation has also been made available. Despite the availability, convenience, and cost effectiveness of online translation (e.g., google translate), there is controversy related to the validity of this approach when used as the sole method of translation. It has been suggested that if one were to consider using an online program, a more valid approach is a hybrid method with traditional translation by experts with high-level degrees in linguistics in combination with an online program (26). Whatever approach is chosen, researchers must ultimately decide on the translation and adaptation procedures that are most appropriate for their scope of work with consideration of time constraints and available resources.

In order to use a HRQOL instrument appropriately in a new country or culture, the instrument must not only possess linguistic equivalence, but must also capture the cultural differences in disease expression and perception of HRQOL (27, 28). We will expand upon this process of “cross cultural adaptation” later in this review.

### Regional and Environmental Differences

An individual's region of origin and environmental context are important considerations during HRQOL assessment. The built environment, defined as the space in which people spend their time in daily life (e.g., home, neighborhood, transportation, or workplace), is closely associated with their health status (29). Seasonal and weather conditions affect physical activity and psychological states (e.g., winter season, unfavorable weather, or decreased sunlight exposure vs. the more positive alternative) (30). Air pollution represented by particulate matter (PM_2.5_) is associated with increased respiratory symptoms and worsened HRQOL (31, 32). There is also evidence to suggest that habitat may influence HRQOL. For example, there are reported differences in HRQOL scores between those in rural vs. urban environments (33–36). These environmental contextual factors may play a role in our interpretation of HRQOL scores amongst different populations and more work is needed to formulate an approach to addressing this issue. Few clinical studies have corrected for the various potential regional and environmental effects on HRQOL, and this is an important area of potential investigation in the future.

### Health Literacy and Diversity of Healthcare Systems

Health literacy is defined as the ability to access, understand, and effectively use health information (37, 38). Patients with low health literacy have less of an understanding about their medical conditions and treatments. This is associated with the potential to worsen health status and disease outcomes (39, 40). A recent study revealed that older age, higher body mass index, residence far from medical institutions, lower monthly income, and lower education levels are associated with a lower health literacy (41). The access to primary care systems and the presence of reliable, understandable, and comprehensive native language medical information websites also contribute to global differences in health literacy (42). The same intervention for a particular chronic disease may be interpreted differently by two patients depending on their comprehension, which may drastically impact patient decision making. Healthcare professionals have made a large effort to improve the impact of low health literacy, including establishment of universal education systems, but many inequalities still exist (42). While mobile health applications may help to enhance interactive patient-provider communication, there is more investigation needed to creatively adapt this technology for use in more remote and resource-limited parts of the world (17, 43–45).

### Race, Ethnicity, Religion, and Spirituality

There is a growing body of literature that reveals the association between race, ethnicity, religion, spirituality and HRQOL. A recent study showed that racial and ethnic differences were associated with differences in HRQOL even within the same community (46). If the prevalence of a certain chronic disease is low in a particular race or ethnic group, the negative impact on HRQOL may become greater (47). A lack of familiarity with a chronic disease in a patient's community may lead to social discrimination, with a negative downstream impact on HRQOL (48). A systematic review focused on the relationship between religiosity/spirituality and quality of life (QOL) in patients with cardiovascular disease found a positive association between mental and emotional well-being, spiritual well-being, intrinsic religiousness, and frequency of church attendance (49). While it is important to recognize that these factors play an important role in HRQOL, there is controversy over the extent to which patients should be subdivided by spiritual and religious beliefs for clinical and epidemiological research (50). In order to address these differences, one potential approach is to focus on the longitudinal relative changes in each individual's HRQOL score, rather than comparing cross-sectional absolute values between different patients, but more work is needed to better define and operationalize this approach.

## Cross Cultural Adaptation

In the past several decades, the measurement of HRQOL has garnered significant attention as an important endpoint in clinical trials and public health research (51). With the increasing number of multi-country, multi-center trials that are conducted in clinical research there is a growing need for HRQOL measures that can be administered in countries with various languages of origin and amongst different cultural groups where disease expression and health-care system usage may vary (52). In order to administer an HRQOL instrument appropriately in a new country or culture, the instrument must not only possess linguistic equivalence, but must also capture the cultural differences in disease expression and perception of HRQOL (27, 28). This allows investigators to collect accurate information about HRQOL of the whole population in one study (when several countries are represented) and to compare results across different studies both nationally and internationally (53). Development of a new PROM is a rigorous and time intensive process (20). It may take years to gather enough data to prove the instrument possesses adequate validity to use in a clinical trial, often with stringent regulatory approval criteria that must be met (54). Rather than develop a brand-new instrument for each distinct language and culture, current practice is to perform “cross cultural adaptation.” This process facilitates the translation of existing and well-validated instruments in a manner that allows the instrument to retain its psychometric properties in a culturally distinct population (55).

There is overwhelming agreement that an instrument should not be simply translated word for word into another linguistic context, as this can compromise the cultural integrity and equivalence of the findings (56). However, there is not a standardized protocol for linguistic validation or cross cultural adaptation, therefore risking poor translation and compromised research data (57, 58).

Several approaches to cross cultural adaptation have been suggested with the goal of maximizing validity and reliability of the instrument that is to be translated into the “target” (or new) language. The Translation and Cultural Adaptation Group (TCA) of the International Society for Pharmacoeconomics and Outcomes Research (ISPOR) task force put forth recommendations for translation and cultural adaptation of PROs in the research community based upon review of the literature and multidisciplinary expert consensus (59). Their recommended approach includes stages of translation and validations testing that require both forward and backwards translation, harmonization that allows for concept equivalence between the source and target language versions of the instrument, review by an expert committee, and cognitive debriefing to assess comprehensibility and cognitive equivalence of the translation by interviewing patients from the target population (60, 61). While the ISPOR task force guidelines provide a rigorous approach to translation, they provide less guidance on further psychometric testing to perform beyond translation and assessment of content validity.

In 1991, the international quality of life assessment (IQOLA) project was established to translate and validate the short-form 36-item health survey (SF-36) (28, 62, 63). The IQOLA project group guidelines encompass a three-stage process that incorporates further psychometric testing; (1) rigorous translation and evaluation process, (2) formal psychometric testing of the assumptions underlying item scoring and construction of multi-item scales, and (3) studies evaluating the equivalence of interpretations across countries (64, 65). Their project with the SF-36 transferred an existing generic HRQOL questionnaire to another culture, a process termed “sequential development”. On the other hand, in 1990s, the World Health Organization (WHO) developed the WHO Quality of Life assessment instrument (WHOQOL) simultaneously in fifteen different centers worldwide (2). This type of approach helps to ensure equivalence of concepts at each stage as the questionnaire is developed in multiple languages at the same time, a process termed “simultaneous development”. In the 1980-1990s, the European Organization for Research and Treatment of Cancer (EORTC) and the EuroQol Group developed the quality of life questionnaire (QLQ-C30), and the EuroQol-5 dimensions (EQ-5D), respectively (66–68). These questionnaires were generated in one language and then forward and backward translated into multiple languages by multinational discussions, a “parallel development” approach. With these historical developments, various HRQOL questionnaire translations are available for clinical trials, daily clinical practice, population studies, and health economic evaluations around the world.

## Cross Cultural Adaptation in ILD

Several patient-reported outcome measures (PROMs) have been adapted for use in ILDs. The PROMs utilized in ILD research and practice are mainly categorized into three groups: (1) disease-specific HRQOL, (2) generic HRQOL, and (3) domain-specific instruments (69). These instruments are ideally chosen as endpoints in research according to the objective of the study and characteristics of the study population. Each of the most common PROMs administered in ILD are at different stages of validation, translation, cross-cultural adaptation, and level of use in clinical trials (Table 1). Here we provide an overview of the current state of cross-cultural adaptation of PROMs in ILD.

**Table 1:** Cross-cultural adaptation and linguistic validation of PROMs in ILD.

**Patient-reported outcome measure**	**Validated IDL**	**Originally development and translations**	**Multi-center/country clinical trial use in IDL**	**MCID**	**References**
**DISEASE-SPECIFIC**					
SGRQ	IPF CTD-ILD	Developed in 1991 English for the UK 170 translations	Yes	IPF: 4–8 CTD-ILD: 4–13	(70–92)
SGRQ-I	IPF	Developed in 2010 English for the UK 1 translation	No	IPF: 4–5	(93–95)
CAT	IPF CTD-ILD	Published in 2009 English for the UK 62 translations	No	IPF: N/A CTD-ILD: 1–4	(96–98)
K-BILD	IPF ILD	Published in 2012 English for the UK 6 translations	Yes	IPF/ILD: 4–8	(83, 99–104)
ATAQ-IPF	IPF	Published in 2010 English for the USA 2 translations	Yes	N/A	(105, 106)
L-IPF (L-PF)	IPF PF-ILD	Published in 2020 English for the USA In translations process	Not yet	Validation process	(107, 108)
PESaM	IPF	Published in 2017 Dutch for Belgium and the Netherlands 1 translation	Not yet	Validation process	(109–111)
CHP-HRQOL	HP	Development and Content Validity Published in 2021 English for the USA Undergoing further validation	Not Yet	Validation process	(112)
**DOMAIN-SPECIFIC**					
**Dyspnea**					
UCSD-SOBQ	IPF CTD-ILD	Developed in 1987 English for the USA 53 translations	Yes	IPF: 8 CTD-ILD: N/A	(80, 81, 83, 84, 87, 91, 92, 113–116)
mMRC	IPF ILD	Modified in 1976, 1986 English for the UK 12 translations	No	N/A	(71, 117, 118)
BDI-TDI	IPF SSc-ILD	Published in 1984 English for the USA 96 translations	Yes	IPF: N/A SSc-ILD: 1.5	(90, 119–121)
**Cough**					
LCQ	IPF	Published in 2003 English for the UK 23 translations	Yes	Chronic cough: 1.3	(91, 122–124)
CQLQ	IPF	Developed in 2002 English for the USA 4 translations	No	IPF: 5	(125)
**Fatigue**					
FAS	IPF Sarcoidosis	Developed in 2003 Dutch for the Netherlands 2 translations	No	IPF: N/A Sarcoidosis: 4	(126–129)
**Anxiety/depression**					
HADS	IPF	Developed in 1983 English for the UK 118 translations	Yes	N/A	(130–133)
**Sleep disorders**					
ESS	IPF	Developed in 1983, and revised in 1997 English for Australia 95 translations	No	N/A	(134–136)
**Generic HRQOL questionnaires**					
SF-36	IPF SSc-ILD	Developed in 1998 (current version) English for the USA 191 translations	Yes	IPF: 2–4 SSc-ILD: N/A	(73, 80, 81, 86, 90, 119, 137–141)
EQ-5D-5L	ILD	Developed in 2011 Dutch for the Netherlands, English for the UK, Finnish for Finland, Norwegian for Norway, Swedish for Sweden 181 translations	Yes (including EQ-5D)	ILD: 0.005–0.095	(142–145)
PROMIS-29	IPF SSc-ILD	Published in 2005 English for the USA 47 translations	Not yet	N/A	(146–148)

*ATAQ-IPF, A Tool to Assess Quality of life in IPF; BDI-TDI, Baseline Dyspnea Index-Transition Dyspnea Index; CAT, Chronic obstructive pulmonary disease Assessment Test; CHP-HRQOL, Chronic Hypersensitivity Pneumonitis Health Related Quality of Life; CQLQ, Cough Quality of Life Questionnaire; CTD-ILD, connective tissue disease associated interstitial lung disease; EQ-5D-5L, EuroQol-5 Dimension-5 Level; ESS, Epworth sleepiness scale; FAS, Fatigue Assessment Scale; GAD-7, Generalized Anxiety Disorder-7; HADS, Hospital Anxiety and Depression Scale; ILD, interstitial lung disease; IPF, idiopathic pulmonary fibrosis; K-BILD, King's Brief Interstitial Lung Disease health status questionnaire; LCQ, Leicester Cough Questionnaire; L-IPF, Living with IPF; L-PF, Living with Pulmonary Fibrosis; MCID, minimal clinically important difference; MFI, multidimensional fatigue inventory; mMRC, modified Medical Research Council dyspnea scale; PESaM, Patient Experiences and Satisfaction with Medication; PF-ILD, progressive fibrosing ILD; PROMIS, Patient Reported Outcome Measurement Information System; SF-36, Short Form-36; SGRQ, St George's Respiratory Questionnaire, SGRQ-I, IPF-specific version of the St George's Respiratory Questionnaire; SSc-ILD, systemic sclerosis related interstitial lung disease; UCSD-SOBQ, University of California San Diego-Shortness of Breath Questionnaire*.

*The number of translations was referred from ePROVIDE^TM^ from MAPI RESEARCH TRUST (https://eprovide.mapi-trust.org) and EQ-5D from EuroQol group (https://euroqol.org)*.

### Disease-Specific HRQOL PROMs

Disease-specific HRQOL PROMs in ILD often provide information about the impact of the patient's lung disease on their quality of life. The St George's Respiratory Questionnaire (SGRQ), which was originally developed for patients with chronic obstructive pulmonary diseases (COPD), is one of the most extensively used PROMs for patients with ILDs (70–92). The SGRQ is relatively well-validated in ILD, however there are concerns regarding the applicability of several of the items to patients with ILD. While the SGRQ length and complicated scoring algorithm may pose some challenges for use in daily clinical practice, it has been translated into a wide range of languages making it a potentially attractive option when conducting multinational studies. The cross-sectional reliability of an IPF-specific version of SGRQ (SGRQ-I), has been reported for patients with IPF, however longitudinal data, language translations, and experiences in clinical trials are limited (93, 94). The COPD Assessment Test (CAT) is a short and simple questionnaire developed for COPD and is reported to correlate well with the SGRQ in IPF and connective tissue disease-associated ILD (CTD-ILD), but experiences in clinical trials is limited (96–98). The King's Brief ILD (K-BILD), is a disease-specific instrument developed in the UK for use in ILD and has been tested in patients with a large number of ILDs (99–102). There is translation and cultural adaptation data for the K-BILD available for several European and South American countries (149, 150), and it is available in multiple languages for use across the globe. Additionally, A tool to Assess the quality of life in Idiopathic Pulmonary Fibrosis (ATAQ-IPF) which was developed initially in the United States to measure HRQOL in Pulmonary Fibrosis has published data on reliability and validity in Chinese patients (cATAQ-IPF) (105, 151).

The Living with IPF questionnaire (L-IPF) (107), developed in the English language, has published initial validation data in a cohort of patients with IPF and has recently expanded applicability as the Living with Pulmonary Fibrosis questionnaire (L-PF) (108). The Patient Experiences and Satisfaction with Medications questionnaire (PESaM) is a unique instrument evaluating patients' expectations, experiences, and satisfaction with disease-modifying drugs (109, 110). This instrument was developed in the Netherlands and provides systematic evaluation of patient experiences and expectations that may allow for improved shared-decision making. For these more newly developed instruments, more data is needed on the applicability across different languages and cultures.

### Generic HRQOL PROMs

Generic HRQOL measures are designed to assess the overall health status across the general population, regardless of a specific type of chronic disease that one may have. Many of these instruments have been well-translated into a wide range of languages and well-validated in various ways as mentioned above. These instruments allow us to compare the health status between patients with different chronic diseases and healthy people. They are valued as key secondary endpoints in many clinical trials.

The SF-36 is the most widely used generic HRQOL measure. The validity of the SF-36 in ILDs has been established since the 1990s, with various studies reporting the cross-sectional and longitudinal validity in IPF, and has been used in many clinical trials of patients with ILD (73, 80, 81, 86, 90, 119, 137–141). As the minimal clinically importance difference (MCID) for the SF-36 in IPF varies depending on the cohort, further global validation studies are required. The EuroQol-5 dimensions 5-level (EQ-5D-5L) is also a well-known and widely-translated generic HRQOL measure. EQ-5D-5L was developed by the EuroQol Group to improve the instrument's sensitivity as compared with the previous version (142, 143). The scores obtained from EQ-5D-5L can be used to calculate quality-adjusted life years (QALY), a generic measure of disease burden. QALY measurements enable investigators to assess both the quality and the quantity of life lived and to examine the value of medical interventions (144). A recent large cohort study demonstrated the construct validity and MCID of EQ-5D-5L in patients with a variety of fibrotic ILD subtypes (145). The Patient-Reported Outcomes Measurement Information System (PROMIS) is a research initiative launched by the National Institutes of Health to develop the PROMs for clinical research and practice across a wide variety of chronic diseases (146). Some studies have shown that PROMIS-29 accurately reflects the deficit in HRQOL of patients with IPF and systemic sclerosis-associated ILD (SSc-ILD), but it is still not widely used in the field of ILD (147, 148).

### Domain-Specific PROMs

Domain-specific PROMs focus heavily on specific symptoms that patients may experience, which in ILD often include dyspnea, cough, fatigue, anxiety/depression, and sleep disturbance. While these PROMs do not measure HRQOL per say, they are important to mention as we know that many of these physical and psychologic symptoms are larger drivers of HRQOL in ILD. Among these, dyspnea and cough are most often assessed in ILD studies.

The University of California San Diego-Shortness of Breath Questionnaire (UCSD-SOBQ), the modified Medical Research Council dyspnea scale (mMRC), the Baseline Dyspnea Index-Transition Dyspnea Index (BDI-TDI), and the dyspnea-12 (D-12) are common questionnaires administered to assess dyspnea in ILD. The UCSD-SOBQ has been administered in different ILD clinical trials and is well-translated in other languages aside from English. The MCID for IPF has been assessed (80, 81, 83, 84, 87, 91, 92, 113–116). The mMRC is a simple and easy tool for use in daily clinical practice and is reported as a useful predictor of mortality. Experience administering the mMRC in clinical trials and the number of linguistic translations is limited (71, 117, 118). The BDI-TDI assesses both baseline and change measures over time. It is well-translated into multiple languages, however there is little reported experience in clinical trials (90, 119–121). The D-12 is a brief and reliable instrument with positive validation data in ILDs but experience in clinical trials and the number of linguistic translations are limited (113, 152, 153).

The Leicester Cough Questionnaire (LCQ) and the Cough Quality of Life Questionnaire (CQLQ) have been used to assess severity, frequency, and impact of cough in patients with ILD. LCQ is a reliable and relatively easy to complete measure, and there is some experience using it in clinical trials. The responsiveness and MCID are not yet reported in ILD (91, 122–124). CQLQ is a comprehensive and responsive measure, and has good cross-sectional validity in IPF, however our experience using this questionnaire in ILD is still limited (125). More studies are needed to assess the validity of cross-culturally adapted versions of these instruments.

## Remaining Gaps and Opportunities

Despite the great strides that have been made to highlight the importance of HRQOL in ILD in the past two decades, there are still many opportunities to internationally and cross culturally improve its measurement. The ILD-specific HRQOL questionnaires (e.g., K-BILD, ATAQ-IPF, or L-IPF/L-PF) are designed to measure the nuanced impacts of ILD on HRQOL more precisely than generic instruments. A limited number of translations and cross-cultural adaptations have been performed on these instruments making them less generalizable for use in a larger international study compared to others that may be less ILD specific, but have been around longer and are more widely established (99–102, 105, 107, 108, 149). For example, questionnaires developed for COPD (e.g., SGRQ) are not specific to ILD, but have a large number of translations and are relatively-well-validated in ILD (70–92). More studies are needed to continue to linguistically validate and cross culturally adapt the new ILD disease-specific instruments. To standardize this process internationally, it will require global consensus and a collaborative approach (95).

There is little information on the international equivalence of the methods we use to validate PROMs, e.g., how we calculate internal consistency, construct validity including correlation with other parameters, and responsiveness. The various global concepts that impact HRQOL have the potential to affect the interpretation of PROMs. These diversities may contribute to different interpretation of the items in a single questionnaire amongst various communities and countries. Although no formal method has been established to address this possibility, subgroup analyses of multinational clinical trials may support the validity of each questionnaire across these communities and nations if similar results are obtained (154–156). We must also recognize that a PROM is ideally chosen to measure a certain outcome based upon the context and objective of the research study. This means that one questionnaire that is deemed appropriate for one trial design may not be the same questionnaire that is ideal for another, even if they are both measuring HRQOL. This adds another layer of complexity for multi-national studies as one must not only choose an instrument that will capture information about HRQOL in multiple languages and cultures, but they must also be comfortable that the instrument is measuring the constructs that are important to answer their particular question.

To date, trials testing medications developed for use in fibrotic ILDs have overwhelmingly targeted the halt of disease progression as reflected by pulmonary function, exercise tolerance, or progression-free survival (82, 103, 115, 157). As disease-specific HRQOL PROMs generally reflect changes in these parameters, these have characteristically been chosen for use in those clinical trials (77, 98, 100). As patient-centered research in ILD expands, future interventions may target the more disease-specific symptoms (e.g., cough, dyspnea, fatigue) (158–160). For these clinical trials, domain-specific PROMs focusing on each symptom may likely be chosen as the primary endpoint and therefore these instruments will need to be adapted for use cross-culturally.

The guidelines for development of PROMs are not internationally unified. Regulatory agencies such as the US Food and Drug Administration (FDA) and the European Medicines Agency (EMA) have released PROM development guidance as we increasingly recognize the importance of including these measures in clinical trials (161, 162). Recent PROMs including the K-BILD and L-IPF/L-PF adhered strictly to their guidelines during the process of developments (99, 107). Although there is no question that these guidelines are well-established and rigorous, it is necessary to verify whether the same methodology can be adapted in non-English speaking countries where there are different cultural components as well as potentially different resources available.

Finally, we need to consider the international inequalities of HRQOL itself. As discussed in this review, many individual factors are closely associated with a patient's health status. In fact, the global burdens of ILD measured by disability-adjusted life years (DALYs), which is calculated as the number of years lost due to disability or early death, are known to greatly vary across the countries (163). The level of HRQOL impairment may differ in each country, even if the disease severity assessed by pulmonary function is the same. Therefore, an understanding of the baseline health status in any individual country is important. If there is a large difference in the baseline health status between groups, then the evaluation of relative change in each individual or group should be considered. Multinational consortia of researchers with expertise in PROMs and who study HRQOL are needed in order to begin to address some of these gaps on an international level.

## Conclusion

HRQOL is an increasingly important end point in ILD amongst patients, clinicians, and researchers alike. As our understanding of the disease and its possible therapies expands, we are rapidly accelerating opportunities for clinical trial conduct across the globe. While we have made great strides in the measurement of HRQOL in ILD, we have many opportunities to improve our measurement across cultures and countries. We have identified several ways in which HRQOL may be interpreted differently across the globe and highlighted potential mechanisms for translation and cross-cultural adaptation of HRQOL PROMs, both in general and in ILD. By recognizing these important differences and working together with our colleagues and patients across the globe we have the opportunity to improve the way we study and report HRQOL which will have a substantial impact on the conduct of multinational studies and interventions in the future.

## Author Contributions

KA and AS contributed equally to the conception and writing of this manuscript. Both authors contributed to the article and approved the submitted version.

## Conflict of Interest

The authors declare that the research was conducted in the absence of any commercial or financial relationships that could be construed as a potential conflict of interest.

## Publisher's Note

All claims expressed in this article are solely those of the authors and do not necessarily represent those of their affiliated organizations, or those of the publisher, the editors and the reviewers. Any product that may be evaluated in this article, or claim that may be made by its manufacturer, is not guaranteed or endorsed by the publisher.

## Footnotes

^1^Population Assessment of Health-Related Quality of Life.
